# Modeling the Relationship Between Tooth Color and Skin Color in Equations to Predict Tooth Color

**DOI:** 10.7759/cureus.26466

**Published:** 2022-06-30

**Authors:** Ryhana Mohammmad Zakir Hiyat Moazam, Zuryati Ab-Ghani, Wan Muhamad Amir W Ahmad, Mohamad Syahrizal Halim, Nafij Bin Jamayet, Matheel AL-Rawas

**Affiliations:** 1 Prosthodontic Unit, School of Dental Sciences, Health Campus, Universiti Sains Malaysia, Kelantan, MYS; 2 Prosthodontic Unit, Hospital Universiti Sains Malaysia, Kelantan, MYS; 3 School of Dental Sciences, Health Campus, Universiti Sains Malaysia, Kelantan, MYS; 4 Conservative Dentistry Unit, School of Dental Sciences, Health Campus, Universiti Sains Malaysia, Kelantan, MYS; 5 Division of Restorative Dentistry, School of Dentistry, International Medical University, Kuala Lumpur, MYS

**Keywords:** skin color, tooth color, tooth color measurement, dental color measurement, digital color measurement, tooth rehabilitation, esthetics dentistry, dental photography

## Abstract

Background

Color selection for discolored teeth or for edentulous patients is a subjective process in dentistry. To the best of the authors’ knowledge, there is no objective method for tooth color selection for edentulous patients. Several studies have investigated the relationship between tooth color and skin color and the ability to use skin color as a guide to select tooth color in dental rehabilitation cases. The objective of this study was to find and model the relationship between tooth color and skin color in equations to be able to predict tooth color by assessing skin color.

Methodology

This study was done on 150 participants with equal gender distribution. Digital photography with soft boxes and strobe lights were used to measure tooth and skin color. Digital photography was performed in a dark room with controlled and measured lighting sources and strength. A calibration board was used to create two calibration profiles in software. All photos were calibrated in Adobe Lightroom software. Then, color measurement was done using Adobe Photoshop software in the Commission International de I’Eclarirage (CIELab) system.

Results

There were positive significant relationships in color spaces L* and a* between tooth color and skin color, and an inverted significant relationship in color space b* between tooth and skin. Three equations with excellent reliability were formulated in this study to predict tooth color by assessing skin color.

Conclusions

The relationship between tooth color and skin color was modeled in three equations. For edentulous patients, this research might be useful in detecting the proper tooth color and transferring the CIELab color data to dental technicians for further processing. Computerized shade matching and prediction dental software may be designed using these equations.

## Introduction

Achieving an aesthetically pleasing smile is among the difficulties dentists must overcome. Harmonizing the surrounding facial structures, such as hair, complexion, and eyes, as well as age, with the color of the teeth was seen as a difficult problem [1,2]. Choosing tooth color is subjective with no set processes or protocols to follow. With the growing awareness and demand for aesthetic dentition, a significant number of studies were conducted to determine what factors could affect the cosmetic of artificial teeth. If there are remaining teeth, selecting tooth color for missing teeth is a straightforward process. However, in cases of edentulous patients, or for patients who need to change their tooth color due to tooth discoloration, skin color may be used as a guide [3].

It has been shown that the color of a person’s teeth is one of the most critical aspects that might influence aesthetic factors [3]. Some studies have investigated the relationship between the color of teeth and some of the facial components. According to a study [4], the color of hair is an unreliable indicator for choosing the color of teeth because hair color is subjected to change more rapidly than tooth color. The study also stated that using the color of the eyes as a reference is questionable because the pupils are relatively small in comparison to the face and are located far from the teeth [4]. A study found a significant inverse association between the color of teeth and the color of skin. Participants who had darker skin had lighter teeth than those who had lighter skin color [5]. Another study [6] came to the same conclusion reporting an association between the color of teeth and the color of skin and eyes.

However, there is still a dearth of knowledge on the relationship between tooth color and skin tone, which has an impact on the ability to select prosthetic teeth [7]. There is currently no specific tooth-skin reference or standard technique that can help dentists objectively match tooth color with skin color. Most of the studies used a subjective visual evaluation and categorization of skin colors, including dark, fair, and medium skin color. Several factors, such as the patient’s age, sex, xerostomia, nutrition, and smoking, as well as dental experience, dentist color blindness, eye exhaustion, and lightening condition, have been reported to influence the visual tooth shade matching [8-10]. In addition, the dentist’s skills and the applied shade guide system can result in diverse outcomes [10]. Therefore, digital instruments, such as spectrophotometers, are more accurate and efficient than visual color matching methods [9,11,12].

There are three null hypotheses of this study. First, there is no significant relationship between tooth color and skin color, sex, and tooth type in color space L*. Second, there is no significant relationship between tooth color and skin color, sex, and tooth type in color space a*. Finally, there is no significant relationship between tooth color and skin color, sex, and tooth type in color space b*.

This study used a reliable and accurate digital color measurement for skin and tooth color. That is to develop a guide or standard technique for tooth color selection regarding skin color, tooth type, and gender. The goal of this study was to assist dentists in making objective measurements of skin color and tooth color. Moreover, to use equations to model this relationship between tooth color and skin color and use these equations to predict tooth color by knowing skin color. The goal of this study was to assist dentists in making objective measurements of skin color and tooth color by modeling the relationship between tooth color and skin color in equations and using these equations to predict tooth color by assessing skin color.

## Materials and methods

Study design and ethical approval

This study is a cross-sectional study. Ethical approval was granted by the Human Research Ethics Committee (USM/JEPeM/ 20110552). All participants signed a consent form for their voluntary participation in the study. There were no conflicts of interest in this study.

Participants

A total of 150 staff and students with equal gender distribution (76 males, 76 females) participated in this study as they were selected by convenience sampling. Participants’ recruitment and data collection took place from April 2021 to June 2021. Informed consent was obtained first. Subsequently, patients were asked to remove all make-up, such as lipstick, and sunscreen. They were also asked to brush their teeth for two minutes with a new toothbrush and toothpaste before data collection. Participants who wore a hijab were requested to cover their heads and shoulders with a gray-colored hijab.

Inclusion and exclusion criteria

The inclusion criteria were Malaysian males and females, systemically healthy, with sound anterior teeth. Participants were excluded from the study if they had discolored teeth (intrinsic or extrinsic), endodontically treated teeth, or necrotic teeth. In addition to participants with missing anterior tooth/teeth, teeth bleaching, or who had undergone a skin tanning procedure, those who were heavy smokers (smoking more than 25 cigarettes a day) [13], heavy tea or caffeine consumers, and those with common skin disorders were also excluded.

Photography settings

For more reliable and accurate results, skin and tooth photography and color measurements were performed under the same circumstances and method with slight differences. Commission International de I'Eclarirage (CIELab) system was used.

A Nikon D90 digital single-lens reflex (DSLR) camera was used for photography. Two lenses were used in this study, one for intraoral photography (Nikon N AF-S MICRO NIKKOR 105 mm 1:2.8 G ED), and another for frontal portrait photography (Nikon DX, AF-S NIKKOR 18-135 mm 1:3.5-56 G ED).

The study was conducted in a dark room. Two light strobes with soft boxes (Lastolite lumen 8 F400) with color temperature ranges between 5,000 K and 6,500 K were used for lighting during photography, including the camera’s own flash. The camera’s own flash was used to trigger the studio lights. The settings of the strobe lights were (ON) Model, (ON) full-modeling bulb, (ON) remote, (ON) beeper, and (1/2) power dial. The lights’ angulations, settings, and distance were fixed during the photography.

A gray cloth (non-reflective) was used for the background behind participants, and a silver light reflector was used in face photography only. The reflector was set on the chest of participants under the participants’ shoulders to reflect the light coming from the softboxes. This is to avoid shadow appearances on participants’ faces.

A ColorChecker Digital SG (semi-gloss) (X-Rite PANTONE, Grand Rapids, MI, USA) calibration board was used for the calibration process with its software (ColorChecker Camera Calibration Software v.2.0). Then, the photos were calibrated in Adobe Lightroom 2020, v.5.RU-EN. Adobe Photoshop 2020, v.5.RU-EN, was used for the color measurement of the photos.

For face photography, the camera settings were set on 1/125 for shutter speed, 10 for aperture (f10), ISO 200, and the white-black balance (W/B) was set on auto. On the other hand, camera settings for tooth photography were set on 1/125 for shutter speed, 20 for aperture (f 20), ISO 200, and the white-black balance (W/B) was set on auto. The format of the photo was RAW.

**Figure 1:**
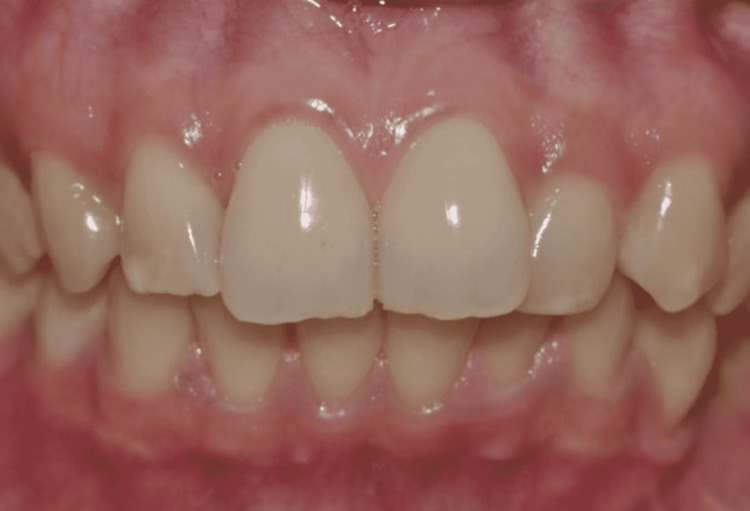
Tooth photography: during this procedure, a cheek retractor was used to retract the cheek.

**Figure 2:**
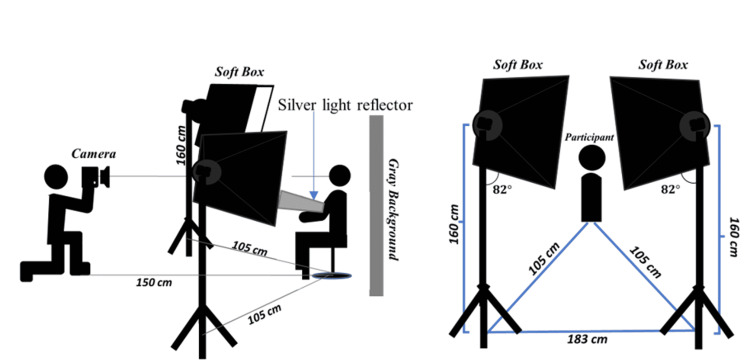
Distance, angulation, and position of the light, camera, and participant for face photography.

**Figure 3:**
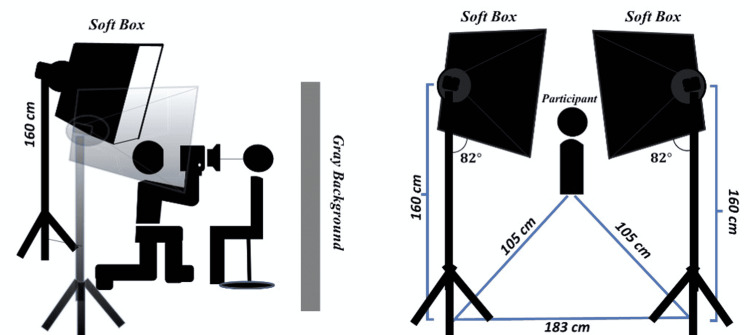
Distance, angulation, and position of the light, camera, and participant for tooth photography.

The distance between camera and tooth depended on the appearance of all maxillary anterior teeth with the mesial side of the first premolar on the digital camera screen (Figure 1). The distance between the camera and the participant’s face was set at 150 cm for face photography. The distance, angulation, and dimensions between softboxes and participants are shown in Figures 2, 3.

Photography and calibration

**Figure 4:**
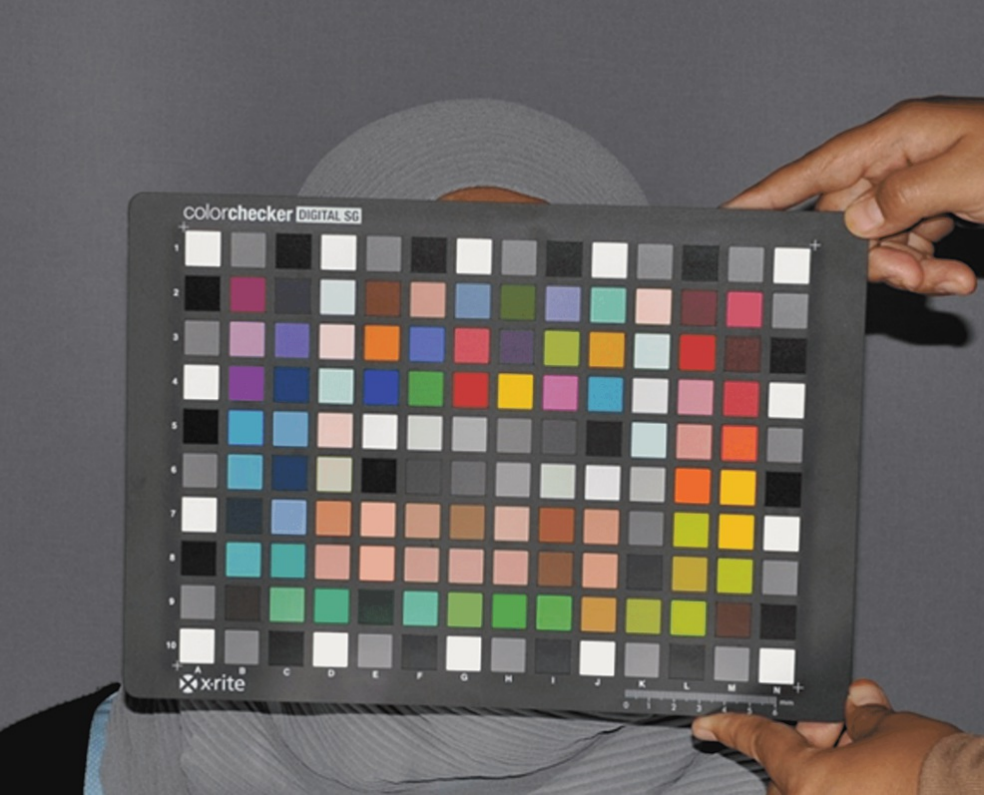
An assistant holding ColorChecker in front of the participant’s face under the settings of face photography. This photo is considered the reference photo for color calibration for face photographs.

First, two photos were taken for the calibration board in front of a participant’s face at 0° viewing geometry. The first photo was taken while using settings for tooth photography. The second photo was taken while using settings for face photography (Figure 4).

These two photos are the references for calibration. After installing these two photos on ColorChecker Camera Calibration Software, two calibration profiles were created. One was to calibrate all tooth photos, and the other was to calibrate all face photos. These profiles were uploaded on Adobe Lightroom.

Then, the research team member took photos of each participant’s face and teeth. The whole procedure took approximately seven minutes for each participant. These photos were calibrated on Adobe Lightroom using the calibrated profiles.

Color measurement

After calibrating all photos, skin and tooth colors were measured using the CIELab system in Adobe Photoshop. Face and teeth photos of all participants were uploaded in Adobe Photoshop. Subsequently, the color picker tool in Adobe Photoshop was used to measure skin and tooth colors from the photos.

For maximum reliability, skin color measurements were done on the participants’ face photos from three points, namely, the chin, right cheek, and forehead [14]. Then, the average was calculated for each color space L*a*b* for each participant’s face color measurement.

Teeth color readings were taken from participants’ tooth photographs from three points (gingival, middle, and incisal) for maxillary central and lateral incisors, and the average was calculated.

To prevent false results, the darkest and lightest areas, such as the zygomatic area or the inclinations of teeth, were avoided in color measurements.

During data collection, all readings from Adobe Photoshop had positive signs only. This indicates that digital camera color reading presented color spaces a and b in the positive axial only. That means colors were presented in the camera reading as follows: L: lightening to darkness (0-100); a: red color (+); b: yellow color (+). There is no presence of green or blue colors.

Reliability

To test the reliability of the equations that modeled the relationship between tooth color and skin color, a correlation test in reliability statistic (intraclass correlation, ICC) test for 30% of data was done. This was done by predicting tooth color by knowing skin color using the equations. Then, the means of two groups (the original tooth color and the predicted tooth color) were tested; p <0.01 was considered statically significant.

Statistical analysis

The data were analyzed in SPSS version 26.0 (IBM Corp., Armonk, NY, USA). In this study, the direct relationship between the dependent variable (camera’s readings for tooth color) and the independent variables (skin color, tooth type, and gender) was analyzed using multiple regression analysis for the three color spaces. One-way analysis of variance (ANOVA) was also used to evaluate the linear significant association between tooth and skin color for the color space a*.

## Results

Regression model for color space L*

The presumption of normality was confirmed. In the regression analysis, the value of R2 was not used because the hypotheses were supported by the p-value, and it was stabilized. Furthermore, a high R2 value does not necessarily indicate that the regression model is adequate because the value of R2 will always increase when a new regression is added, even if the outcomes were less acceptable regarding statistical significance and stability [15].

The correlation test showed that the relationship between the two groups was significant at p <0.01. The ICC value for average measures was 0.993 and 0.985 for single means. According to a book [16], excellent reliability scored in a correlation between 0.81 and 1.00. Therefore, the equations used to predict tooth color have excellent reliability.

Table 1 shows a significant relationship between tooth color and skin color (βskin = 0.02; 95% confidence interval (CI) [-0.00, 0.05]; p = 0.08), (βsex = 0.69; 95% CI [0.31, 1.08]; p < 0.01), and (βtooth type = -5.35; 95% CI [-5.69, -5.01]; p < 0.01). The relationship was also significant between tooth color and sex (p < 0.05) and between tooth color and tooth type (p < 0.05), as shown in Table 1. Therefore, the null hypotheses were rejected.

**Table 1:** Regression analysis between tooth color, skin color, sex, and tooth type for each color space. Dependent variable: tooth reading. Normality Assumption is fulfilled. *Significant at the level 0.05; **Significant at the level 0.25. CI: confidence interval; VIF: variance inflation factor

	Model	Unstandardized coefficients		T	P-value	95.0% CI for B	Collinearity statistics	
Color space		B	Std. Error				Tolerance	VIF
L	(Constant)	70.22	0.68	104.03	0.00*	(68.90, 71.55)		
	Skin reading	0.02	0.01	1.77	0.08**	(-0.00, 0.05)	0.78	1.28
	Sex	0.69	0.20	3.55	0.00*	(0.31, 1.08)	0.78	1.28
	Tooth type	-5.35	0.17	-31.02	0.00*	(-5.69, -5.01)	1.00	1.00
a	(Constant)	3.33	0.41	8.18	0.00*	(2.53, 4.13)		
	Skin reading	0.01	0.02	0.42	0.68	(-0.03, 0.04)	0.99	1.01
	Sex	-0.21	0.09	-2.36	0.02*	(-0.38, -0.04)	0.99	1.01
	Tooth type	1.57	0.09	17.75	0.00*	(1.39, 1.74)	1.00	1.00
b	(Constant)	15.11	0.76	19.88	0.00*	(13.62, 16.60)		
	Skin reading	-0.08	0.02	-3.71	0.00*	(-0.12, -0.04)	0.89	1.12
	Sex	-0.31	0.17	-1.78	0.08**	(-0.64, 0.03)	0.89	1.12
	Tooth type	0.74	0.16	4.56	0.00*	(0.42, 1.06)	1.00	1.00

This means that as skin color, gender, and tooth type vary, so will tooth color. The link between tooth color, skin color, and sex is direct (βskin = 0.02) and (βsex = 0.69), respectively, as they have positive signs. However, due to the negative sign of the relationship between tooth type and tooth color, the relationship is inversed (βtooth type = -5.35). The equations to predict tooth color for the color space L* is as follows: Tooth color L* = 70.22 + (0.02 (skin color L)) + 0.69 (sex) + (-5.35) (tooth type).

Regression model for color space a*

There was no significant relationship between tooth color and skin color in color space a* with p = 0.68 (βskin = 0.01; 95% CI [-0.03, 0.04]; p = 0.68) (Table 1). Therefore, the null hypothesis here was accepted.

It can also be seen from Table 1 that the relationships between tooth color and sex (βsex = -0.21; 95% CI [-0.38, -0.04]; p = 0.02) and between tooth color and tooth type (βtooth type = 1.57; 95% CI [1.39, 1.74]; p < 0.01) were significant. Therefore, these null hypotheses were rejected.

However, by considering that the regression analysis showed no significant relationship between tooth color and skin color in the color space a*, the ANOVA test was done (Table 2). The ANOVA test showed that the linear relationship between tooth color with skin color, sex, and tooth type (F(3, 2380) = [87.527]; p < 0.05) was significant. Therefore, multiple regression analysis was done on color space a* for tooth color as a dependent variable with sex and tooth type as independent variables without skin color, as shown in Table 3. The equation to predict tooth color for color space a* without skin color is as follows: Tooth color a* = 3.46 + (-0.195 sex) + 1.57 tooth type.

**Table 2:** ANOVA test for the relationship between tooth color with skin color, sex, and tooth type for the color space a*. a: Dependent variable: tooth color; b: Predictors: (constant), tooth type, sex, skin color.

	df	Mean square	F	P-value
Regression	3	498.163	87.527	0.00^b^
Residual	2,380	5.692		
Total	2,383			

**Table 3:** Regression analysis of tooth color using the camera in color space a* without skin color. Dependent variable: tooth reading. Normality assumption is fulfilled. *Significant at the level of 0.05. CI: confidence interval; VIF: variance inflation factor

Color space a	Unstandardized coefficients		t	P-value	95.0% CI for B	Collinearity statistics	
	B	Std. Error				Tolerance	VIF
(Constant)	3.46	0.19	18.09	0.00*	(3.09, 3.84)		
Sex	-0.195	0.09	-2.22	0.03*	(-0.37, -0.02)	1.00	1.00
Tooth type	1.57	0.09	17.85	0.00*	(1.40, 1.74)	1.00	1.00

Regression model for color space b*

As shown in Table 1, there was an inverse significant relationship between tooth color and skin color (βskin = -0.08; 95% CI [-0.12, -0.04]; p < 0.05), between tooth color and sex (βsex = -0.31; 95% CI [-0.64, 0.03]; p = 0.08), and between tooth color and tooth type (βtooth type = 0.74; 95% CI [0.42, 1.06]; p < 0.05) in color space b*. This indicates that changing skin color, sex, and tooth type changes will cause a change in tooth color. Therefore, the null hypotheses were rejected. The equations to predict tooth color for this study for the color space b* is as follows: Tooth color b* = 15.11 + ((-0.08) (skin color b)) + (-0.31 sex) + 0.74 tooth type.

## Discussion

Three equations modeled the relationship between tooth color and skin color in this study that could be used for tooth color prediction in cases of dental rehabilitation for edentulous patients or patients with tooth discoloration. To the authors’ knowledge, until now, no study has modeled the relationship between skin color and tooth color in equations to predict tooth color.

Some previous studies that investigated the relationship between tooth color and skin color used conventional shade matching with a makeup skin color chart/shades [1,7,17], or Fitzpatrick skin type test [18], and a conventional tooth color selection using tooth shade tabs. These studies concentrated on and described color according to the relationship between the lightness (value) of the tooth with the lightness (value) of the skin. Therefore, there were no sufficient results on yellow/blue and red/green colors. Furthermore, conventional color matching using the naked eye is subjective as it is subjected to confounding factors [19,20]. Therefore, the results of using the naked eye for tooth color measurement are subject to bias [19,20].

Using digital methods, more conclusive, detailed, and reliable results can be obtained [21-24]. Hence, studies have used digital methods to measure tooth color and skin color [12,25]. However, these studies used two different devices to measure tooth and skin colors. As these studies used spectrophotometers to measure tooth color and digital photography to measure skin color. A study [12] found an inverse significant relationship between tooth and skin color. Another study [25] found that skin color space L* and a* readings were insufficient for describing L* and a* readings of teeth.

As light and calibration control the measurement of color, hence, using different devices with different calibration systems and lighting conditions, a device for measuring skin color, and another device for tooth color measurement, would make the measurement unreliable.

To control these parameters in this study, teeth and face photographs were obtained in a dark room with standardized and fixed light devices, sources, and settings. Spectrophotometers were not included in this study because of the extremely high cost of the skin spectrophotometers. This may limit the application, repetition, and development of this study in future dental clinical tests or clinical applications due to its high cost.

## Conclusions

The relationship between tooth color and skin color was modeled in this study in three equations to predict tooth color CIEL*a*b* values by assessing the skin CIEL*a*b* values, gender, and tooth type. This is extremely beneficial for edentulous patients or for discolored teeth. Moreover, it would help researchers to develop these equations to use in artificial intelligence. These equations can also be used to program dental software for digital shade matching and predicting. Finally, it can also help dentists in the clinic to predict tooth color for their edentulous patients and transfer patients’ tooth CIEL*a*b* color to dental technicians.
